# Left bundle branch area pacing: Electrocardiographic features

**DOI:** 10.1002/joa3.12610

**Published:** 2021-08-07

**Authors:** Asit Das, Suman Chatterjeet Das, Aniruddha Mandal

**Affiliations:** ^1^ Department of Cardiology IPGME&R and SSKM Hospital Kolkata India

**Keywords:** His bundle, left bundle branch area, physiological pacing

## Abstract

**Background:**

Left bundle branch (LBB) area pacing emerged as a promising alternative to His bundle (HB) pacing in difficult cases of physiological pacing and failed cases of cardiac resynchronization. So, it is important to understand ECG features of LBB area pacing in various subsets of patients.

**Objective:**

We wanted to find out different morphological patterns and characteristic ECG features of LBB area pacing.

**Method:**

Medtronic 3830 pacing lead was used to pierce the interventricular septum 1‐2 cm distal towards the RV cavity to a previously placed electrophysiology catheter at distal HB region to reach the LBB area in the right anterior oblique (RAO) 30 degree projection. We observed paced QRS morphology in lead V1 and paced QRS duration.

**Results:**

We have analyzed ECG features of 60 patients who had undergone LBB area pacing and 60 patients with RV apical pacing. LBB area pacing resulted in narrower‐paced QRS complex than conventional RV apical pacing. In patients with baseline LBBB QRS shortening from LBB area pacing was more in comparison to patients with RBBB (34.45 ± 8.07 ms vs 19.78 ± 10.24 ms, *P* value .004). Paced QRS morphological pattern in lead V1 was most commonly qR pattern followed by Qr pattern.

**Conclusions:**

LBB area pacing results in narrower‐paced QRS duration than RV apical pacing. The morphological pattern is most commonly a qR or Qr pattern in lead V1. Patients with baseline RBBB showed lesser paced QRS shortening in comparison to patients with baseline LBBB.

## Graphical abstract

Left bundle branch area pacing results in narrower paced QRS duration than right ventricular apical pacing with most commonly qR or Qr pattern in lead V1. Patients with baseline right bundle branch block showed lesser paced QRS shortening in comparison to patients with baseline left bundle branch block.

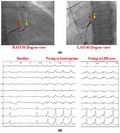

## 1 INTRODUCTION

Pacing from right ventricular (RV) apex results in slow myocyte‐to‐myocyte propagation of the electrical activation wavefront throughout both the ventricles. As a result, surface electrocardiograms (ECG) exhibit a wide QRS complex and left bundle branch block pattern, characteristic of electrical dyssynchrony. This asynchronous electrical activation leads to asynchronous mechanical contraction which induces a spectrum of systolic and diastolic hemodynamic abnormalities in some patients.^1^, ^2^ Several pacing sites inside the RV tried as a better alternative pacing site. Permanent His bundle (HB) pacing (selective or non‐selective) was found to be the most physiological as it did not induce ventricular dyssynchrony (electrical or mechanical).^3^, ^4^ Left bundle branch (LBB) area emerged as an alternative to HB in difficult and failed cases of physiological pacing.^5^ Moreover, LBB area pacing also revealed its potentials as an effective therapy for cardiac resynchronization.^6^ So, it is important to understand ECG features of LBB area pacing in various subsets of patients.

## 2 METHODS

In our hospitals, from 1st January 2019 till 31st December 2019 we have analyzed ECG features of patients who had undergone successful LBB area pacing. We recruited all the patients who had undergone permanent pacemaker implantation during this period alternately to either LBB area pacing or RV apical pacing (control group) in consecutive 150 patients (simple randomization). Based on the baseline QRS duration we grouped all the patients into 3 groups before pacemaker implantation: (a) narrow (≤130 ms) base‐line QRS complex, (b) wide (>130 ms) baseline QRS with left bundle branch block (LBBB), and (c) wide baseline QRS non‐LBBB. The non‐LBBB group included right bundle branch block (RBBB) with either fascicular block (anterior or posterior hemiblock) or IVCD. All patients gave written consent agreeing to the implantation procedure, and this study was approved by the hospital's institutional review board.

**FIGURE 1:**
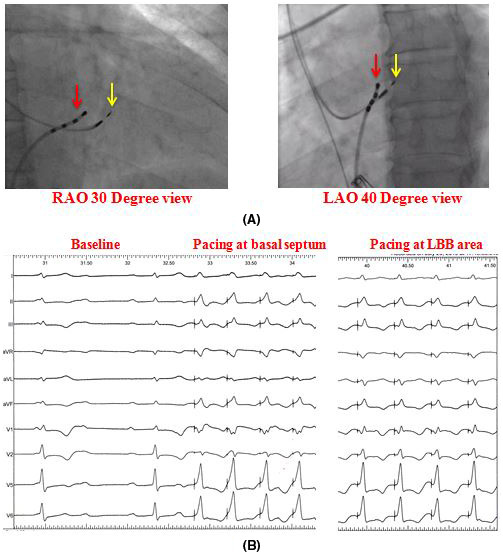
(A) Fluoroscopic RAO 30 degree and LAO 40 degree projection showing relative position of the pacing lead (yellow arrow) and the His Bundle catheter, (B) From left to right showed baseline ECG of complete AV block with narrow QRS complex, unipolar pacing at basal septum before penetration and unipolar pacing at LBB area

Under local anesthesia with the coverage of intravenous antibiotics access to the left subclavian vein was done and the pacemaker pocket was prepared with blunt dissection in left infra‐clavicular region in all patients. In LBB area pacing a select secure (Medtronic, model 3830) pacing lead was positioned 1‐2 cm distal towards the RV cavity to the previously placed electrophysiology (EP) catheter (quadripolar catheter with 2‐5‐2 mm electrode spacing) at distal HB region (characterized by a typical triphasic signal with smaller “a,” sharp H and bigger “V”) catheter in the right anterior oblique (RAO) 30 degree projection (Figure 1A). To avoid pinning of the septal leaflet of tricuspid valve the lead‐sheath assembly was first advanced to the RV apex and then it was withdrawn to the desired location. Twelve lead ECG and the intra‐cardiac electrograms were continuously recorded in an EP recording system (Labsystem Pro EP recording system, Boston Scientific). Baseline AH and HV interval were noted at the annulus in all cases of LBB area pacing by the same quadripolar EP catheter before it was positioned into the distal HB region. Unipolar pacing through the lead tip was performed in this basal part of the septum to identify the ideal site of penetration where the paced QRS complex morphology appeared as “W” pattern (notch in the nadir) and/or paced QRS duration of less than 145 ms in lead (Figure 1B). Then the interventricular septum was penetrated with clockwise rotation on the lead body as described by Zhang et al to reach the left side of the septum where the paced QRS in lead V1 showed RBBB (rightward shift of the notch in lead V1) pattern (Figure 1B).^7^ Specific attention was given to identify any LBB potential recorded from the pacing lead tip. The definition of successful LBB area pacing was the presence of any two of the three criteria: (a) Narrow‐paced QRS complex (≤130 ms), (b) RBBB morphological pattern of the paced QRS complex in lead V1, and (c) short peak LV activation time (pLVAT) (≤90 ms). In cases where we could not get success, we accepted the septal position of the lead for ventricular pacing and excluded them from the study.

In RV apical pacing ventricular pacing lead was positioned at the RV apex with the help of a simple curved stylet. All patients received a dual‐chamber pacemaker with an atrial lead positioned at the right atrial appendage. After positioning, the leads were checked for parameters and stability. Once satisfied the leads were connected to the pulse generator and positioned in the preformed pocket. For patients with failed CRT, the LBB area pacing lead was connected to the LV port of the CRT device and RV lead output was programmed to sub‐threshold level. The wound was closed after achieving proper hemostasis. ECG‐gated AV optimization was done in all patients before discharge to achieve the narrowest paced QRS complex. We observed a change in paced QRS duration (from the intrinsicoid deflection in lead V1/V2 to the end of QRS complex) and QRS duration shortening (base‐line QRS duration minus paced QRS duration) if any. Local ventricular myocardial depolarization time was measured as the interval from pacing stimulus to the peak of R wave in lead V4‐6 in high (5 V) output and the highest value was termed as pLVAT. All the measurements were done by 2 authors independently (AD and SC). In case of discrepancy of 2 observers, the highest value was taken. We looked for paced QRS morphology in lead V1 specifically for qR, Qr, rSR’ or rSr’, M‐shaped, or monophasic R wave.

## 3 RESULTS

In our hospitals, in a period from 1 January 2019 till 31 July 2020 we have analyzed ECG features of 60 patients with successful LBB area pacing (case group) (in 15 cases we failed to achieve successful LBB area pacing; success rate 80%) and 75 patients with RV apical pacing (control group). Table 1 summarizes the indications for pacing in two groups and associated comorbidities. Among these 60 successful LBB area pacing patients we could record LBB potential in only 32 cases (60.17%). In the LBB area pacing group we had 20 patients with narrow (≤130 ms) baseline QRS complex, 40 patients with wide (>130 ms) baseline QRS complex (22 patients with LBBB including 6 patients with failed CRT, and 18 patients with RBBB with either anterior or posterior fascicular block). We did not have any cases of interventricular conduction delay. The indication for pacing in the LBB area pacing group in patients with baseline narrow QRS complex was symptomatic complete heart block (CHB) in 8 (40%) patients, 2:1 atrioventricular (AV) block in 12 (60%) patients. Pacemaker implantation in LBB area pacing in LBBB group of patients was for symptomatic (syncope) LBBB in 4 patients (18.1%), symptomatic LBBB with prolonged PR interval in 10 (45.5%) patients, LBBB with CHB in 2 (9.1%) patients and for failed CRT in 6 (27.3%) patients. The indications for pacing in the non‐LBBB group were symptomatic bifascicular block (RBBB with either anterior or posterior fascicular block) in 8 (44.4%) patients, tri‐fascicular (bifascicular block with prolonged PR interval) in 8 (44.4%) patients, and RBBB with complete heart block in 2 (11.1%). In RV apical pacing group, we had 21 patients with narrow baseline QRS complex, 19 patients with LBBB, and 20 patients with RBBB with either anterior or posterior fascicular block (8 patients had additional PR interval of more than 200 ms). Both the LBB area pacing group and RV apical pacing group had similar demographic profile (age 63.21 ± 8.29 years vs 64.36 ± 9.91 years; male: female—3:5 vs 5:6). The three groups of patients in the LBB area pacing group had also a similar demographic profile (age—62.80 ± 8.79 years vs 60.27 ± 10.42 years vs 65.56 ± 7.92 years and male: female—1:1 vs 5:7 vs 4:5, respectively). Overall 20 (16.7%) patients had diabetes, 24 (20%) patients had hypertension and 12 (10%) patients had both diabetes and hypertension. Table 2 provides important statistical information of these three groups of patients in the LBB area pacing group. The mean baseline QRS duration was similar in both LBB area pacing and RV apical pacing group (130.3 ± 12.8 ms vs 132.3 ± 12.0 ms, respectively, *P* value is .45). In the LBB pacing group the mean baseline QRS duration in patients with narrow QRS group (group A) was 98.9 (±6.5) ms, in the LBBB group (group B) was 148.4 (±6.6) ms, and in non‐LBBB with wide baseline QRS complex group (group C) was 140.6 (±6.3) ms The patients in LBBB and non‐LBBB wide QRS had similar baseline AH interval (119.1 ± 12.4 ms vs 107.0 ± 19.9 ms, respectively, *P* value .17) and HV interval (57.4 ± 9.0 ms vs 65.2 ± 12.3 ms, respectively, *P* value .086). However, patients with the narrow baseline QRS complex in comparison to these two groups had significantly shorter AH (70.7 ± 8.6 ms, *P* value <.001 and <.001, respectively) and HV (46.3 ± 4.3 ms, *P* value .002 and <.001, respectively) intervals. Paced QRS duration was significantly shorter in the LBB area pacing group than RV apical pacing group (116.2 ± 7.1 ms vs 135.1 ± 9.2 ms, respectively, *P* value is <.001). In the LBB area pacing group the paced QRS duration in the narrow baseline QRS group was 104.8 ± 12.3 ms with an increase in QRS duration of 5.9 ± 11.9 ms. Paced QRS duration in the LBBB group was 113.9 ± 5.5 ms with a reduction in QRS duration of 34.5 ± 8.1 ms. The paced QRS duration in the non‐LBBB wide baseline QRS (RBBB) group was 120.8 ± 6.0 ms with the reduction in QRS duration of 19.8 ± 10.2 ms. In patients with baseline LBBB QRS shortening from LBB area pacing was more in comparison to patients with RBBB (34.5 ± 8.1 ms vs 19.8 ± 10.2 ms; *P* value .004). Paced QRS morphological pattern in lead V1 (Table 3) was most commonly qR pattern (in 24 patients 40.07%) followed by Qr pattern (in 20 patients 36.67%). Less commonly QS and rSr’ pattern in lead V1 was seen in 8 (13.33%) and 6 (10%) patients, respectively. No pattern was specific for any of these three groups of patients. The group‐wise distribution of different patterns of QRS morphology in lead V1 during LBB area pacing is listed in Table 3. However, pLVAT was similar in 3 groups: in narrow baseline QRS group was 73.2 ± 4.9 ms, in the LBBB group was 78.2 ± 5.0 ms, in RBBB group was 79.8 ± 4.5 ms. Stimulus to the beginning of the QRS was identical in 3 groups: 17.1 ± 9.2 ms vs 19.4 ± 10.1 ms vs 13.7 ± 10.3 ms, respectively. During positioning the lead at the LBB area 2 patients developed transient complete heart block in the LBBB case group and 1 patient developed RBBB in the narrow QRS case group.

**TABLE 1:** Clinical characteristics of the patients

	LBB area pacing (n = 60)	RV apical pacing (n = 75)
Comorbidities		
DM	9 (15%)	13 (17.3%)
HTN	8 (13.3%)	11 (14.7%)
Smoker	12 (20%)	20 (26.7%)
CAD	0	15 (20%)
Indications for pacing		
Sinus node disease	6 (10%)	8 (10.7%)
AV node disease	14 (23.3%)	15 (20%)
Bundle branch block	40 (67.7%)	52 (69.3%)

Abbreviations: AV, atrioventricular; CAD, coronary artery disease; DM, diabetes mellitus; HTN, hypertension; LBB, left bundle branch; RV, right ventricle.

**TABLE 2:** Major statistical information of the patients

	GROUP								
	Group A (narrow baseline QRS complex		Group B (baseline LBBB)		Group C (baseline non LBBB wide QRS)		*P* value		
	Mean	SD	Mean	SD	Mean	SD	A vs B	A vs C	B vs C
Age (years)	62.80	8.79	60.27	10.42	65.56	7.92	.751	.512	.402
Baseline QRS duration (ms)	98.90	6.51	148.36	6.62	140.56	6.29	<.001	<.001	.018
AH (ms)	70.70	8.56	119.09	12.38	107.00	19.86	<.001	<.001	.171
HV (ms)	46.30	4.32	57.36	9.01	65.22	12.25	.002	<.001	.086
Post pacing QRS duration (ms)	104.80	12.26	113.91	5.52	120.78	6.04	.048	.007	.029
PLVAT (ms)	73.20	4.85	78.18	5.02	79.78	4.52	.030	.009	.353
Stimulus to QRS (ms)	17.10	9.24	29.36	10.10	23.67	10.32	.301	.380	.090
Decrease in QRS (ms)	5.90	11.87	−34.45	8.07	−19.78	10.24	<.001	.001	.004

Abbreviations: LBBB, left bundle branch block; PLVAT, peak left ventricular activation time.

**TABLE 3:** Morphological pattern of the paced QRS complex in lead V1

	GROUP			Total	*P* value		
	GROUP A	GROUP B	GROUP C		A vs B	A vs C	B vs C
PATTERN							
Qr	8 (40)	6 (27.27)	8 (44.44)	22 (36.67)	.900	.836	.590
qR	8 (40)	12 (54.55)	4 (22.22)	24 (40)			
rSr'	2 (10)	2 (9.09)	2 (11.11)	6 (10)			
QS	2 (10)	2 (9.09)	4 (22.22)	8 (13.33)			

## 4 DISCUSSION

RV apical pacing results in abnormal late activation of the lateral wall of the LV because of a differential muscle strain and fiber shortening resulting from which in turn leads to increased myocardial work and oxygen consumption. These changes in cardiac hemodynamics cause LV cellular abnormalities (both at a gross and ultrastructural level) may lead to ventricular remodeling which is associated with a higher risk of development of LV systolic dysfunction, heart failure, and atrial fibrillation.^2^ Permanent, selective, HB pacing was supposed to be the most physiological form of ventricular pacing which replicates the normal activation of the interventricular conduction system.^8^ The recent AHA/ACC/HRS guideline on management of bradycardia recommended HBP as a class IIa indication in patients with AV block who have an indication for permanent pacing with an LVEF between 36% and 50%.^9^ Huang and his colleagues were trying to do HB pacing in a case of failed CRT by mapping different location around HB and discovered the effects of LBB area pacing.^5^ In this case, LBBB correction with LBB area pacing manifested as RBBB. With AV optimization paced QRS gradually narrowed and normalized. The pacing spike in QRS interval was 34 ms. In our study, we found a spike in QRS interval in the LBBB group of 29.36 ± 10.10 ms. Zhang et al found that LBB area pacing is a very effective method of cardiac resynchronization in patients with heart failure and ventricular dyssynchrony caused by LBBB.^6^ In their study, the paced QRS duration and pLVAT were 147.91 ± 16.51 ms and 82.36 ± 13.12 ms. In our study, the paced QRS duration and pLVAT in the LBBB group were 113.91 ± 5.52 ms and 78.18 ± 5.02 ms, respectively. However, we have not done any subgroup analysis for the patients with LBBB with failed CRT because of very low sample volume. An earlier similar study by Chen et al have compared ECG features of LBB area pacing with conventional RV apical pacing and found that LBB area pacing results in much narrower paced QRS (111.85 ± 10.77 ms vs 154.80 ± 9.85 ms) and potentially better synchronization of the LV than conventional RV pacing.^10^ In addition, there was no difference in paced QRS duration between the patients with recorded LBB potential in sinus rhythm and patients without it. In our study, we also found a significantly narrow paced QRS complex in the LBB area pacing group in comparison to RV apical pacing group (116.2 ± 7.1 ms vs 135.1 ± 9.2 ms, respectively, *P* value is <.001). Gao and his colleagues compared ECG features of LBB area pacing with native RBBB and showed that the LBB area pacing‐induced RBBB pattern is distinctly different from typical RBBB.^11^ The majority of the patients showed a Qr/qR pattern in lead V1 with significantly shorter QRS duration with shorter R’ wave duration. They also commented that the integrity of the bundle branch conduction, rather than the presence of an LBB potential, had the most significant impact on the characteristics of the ECG during LBB area pacing. In our study, the majority of the patients with LBB area pacing showed qR and Qr patterns (40% and 36.67%, respectively).

### 4.1 Ventricular activation

**FIGURE 2:**
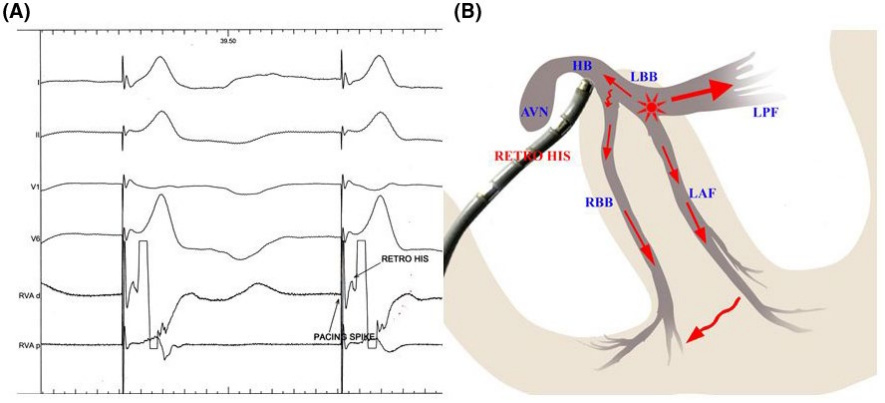
Ventricular activation during LBB area pacing: LV depolarization is rapid as a result of rapid downstream stimulation via native conduction system from the pacing site. RV gets depolarized by 2 wave‐fronts: (a) slow conduction wave from the LV through septal myocardium, and (b) from the pacing site conduction wave goes retrogradely through the native conduction system to reach the distal HB where it is recorded as “retro” His in a prepositioned EP catheter. From there the impulse reaches the RBB through slower conducting transverse connections (may be slower conducting sometimes). Although relative contribution of each one is unknown. Now the impulse reaches the RV myocardium by rapid conduction through the native RBB. Solid red arrow indicates faster conduction and curved red arrow indicates slower conduction. HB, His bundle; LBB, left bundle branch; LV, left ventricle; RBB, right bundle branch; RV, right ventricle

LBB area pacing resulted in narrow (<130 ms) paced QRS complex with an RBBB pattern in lead V1 and short isoelectric stimulus–QRS intervals at low outputs.^10^ LBB area pacing generated two conduction wavefronts, one stimulation wavefront conducted anterogradely from the pacing site to the Purkinje network and subsequently activating left ventricular myocardium, and the second wavefront conducted retrogradely to reach the bifurcation point of HB (distal HB) where it was recorded as the retrograde His potential (in the previously positioned EP catheter) (Figure 2). From there this retrograde conduction wave reach the RBB through some slowly conducting transverse connections (as the bundle branches are insulated and separated from each other by layers of fibro‐connective tissue and already predestined in the His bundle, the transverse connections are required to inter‐connect them). Now, this wavefront recruits RBB and passes downward to the Purkinje network of the RV. In patients with normal bundle branch conduction (ie, normal retrograde conduction in the LBB and intact RBB), 2 conduction pathways were presumed to participate simultaneously in the RV activation: rapid conduction through the RBB and slow left‐to‐right trans‐septal myocardial conduction. Thus, the narrow QRS duration during LBB area pacing might result not only from the rapid LV activation through the LBB (with normal downstream conduction) but also from the rapid RV activation involving conduction through the RBB. So, the paced QRS duration was dependent on the integrity of bundle branch conduction and the extent of biventricular synchronization.

### 4.2 Paced QRS morphology

**FIGURE 3:**
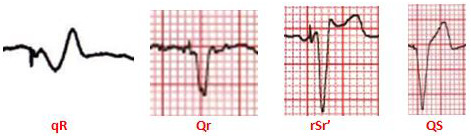
Different paced QRS morphologies in lead V1 during LBB area pacing

The RBBB morphology resulted from LBB area pacing was distinctly different from typical RBBB. The rapid conduction through the RBB could lessen the degree of RV delay and contribute to the narrowing of the terminal R’ wave in lead V1. The ventricular activation during LBB area pacing in patients with intact bundle branch conduction was biphasic with the initial vector directed posteriorly and towards left, appearing as a Q or q wave in lead V1 on the surface ECG, followed by a synchronized rightward vector formed the narrow terminal R’ wave in lead V1 (Figure 3). The LBB area pacing ECG typically displayed a biphasic qR or Qr morphology without an initial r wave in lead V1, in contrast to the rsR’ pattern typically during RBBB.

### 4.3 AV optimization

**FIGURE 4:**
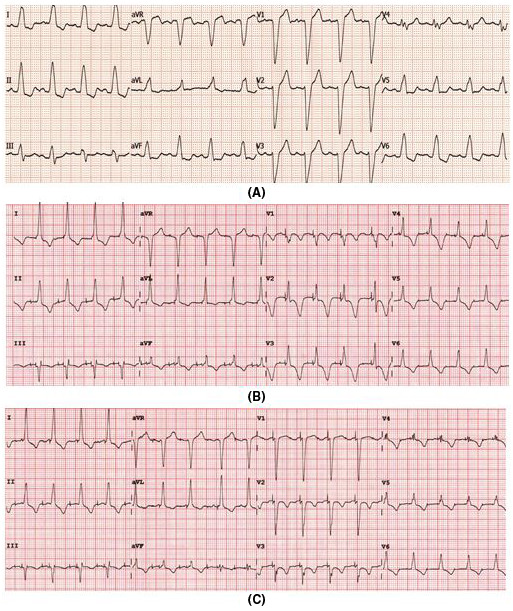
(A) Baseline ECG showed LBBB with QRS duration of 145 ms, (B) Dual‐chamber pacing with ventricular pacing from LBB area with a short AV interval showing QRS duration of 110 ms, (C) Dual‐chamber pacing in the same patient with an optimum AV delay showed paced QRS interval of 100 ms with LBBB corrected without overt RBBB

Adjusting AV delay has an important impact on the correction of LBBB. To short AV delay results in an RBBB pattern and too long AV delay allows intrinsic conduction resulting in LBBB. Pacing at optimum AV delay results in correction of LBBB at the same time avoid overt RBBB pattern because of fusion of LV stimulation wavefront of paced rhythm and RV stimulation of intrinsic conduction wavefront (Figure 4). However, in patients with unreliable AV conduction fusion is not possible. So, further narrowing of paced QRS complex with optimum AV delay by fusion of the paced LV activation wavefront and RV intrinsic conduction does not happen. Moreover, LBB area pacing was more often used in patients with complete AV block in whom fusion seemed to be difficult.

### 4.4 Axis

**FIGURE 5:**
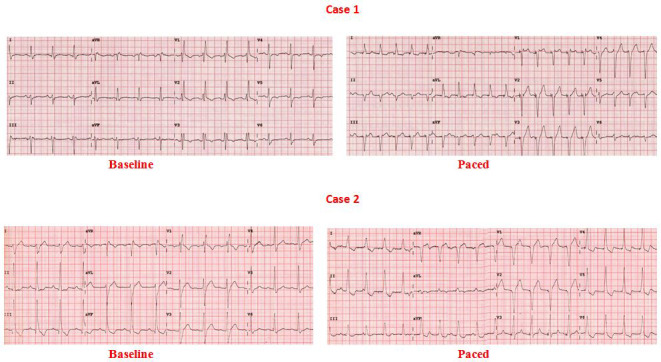
Effects of LBB area pacing in 2 patients with baseline RBBB. In case 1 left anterior hemiblock persisted after LBB area pacing which indicated the probable location of the pacing lead in the left posterior fascicle. In case 2 left posterior hemiblock got corrected which indicated the probable lead location in the main left bundle branch or in the posterior fascicle

Anatomically much wider left posterior fascicle made it susceptible for pacing lead attachment to it instead of main left bundle branch while doing LBB area pacing. Then it results in an RBBB pattern in lead V1 accompanied by a left anterior fascicle delay pattern. When the pacing lead was positioned in the main LBB pacing resulted in an RBBB pattern in lead V1 with the normal axis. Figure 5 demonstrates two cases of pacing at left posterior fascicle. So, if paced at the region of left posterior fascicle LBB area pacing results in leftward axis, and if paced at the main LBB it results in normal axis.

### 4.5 Intervals

**FIGURE 6:**
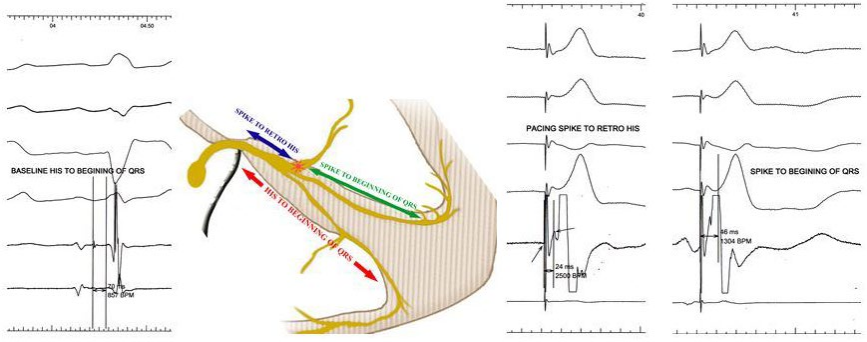
A method to prove engagement of left bundle branch in patients with LBBB. Usually, in patients with baseline, LBBB the LBB potential is not recordable from pacing lead tip positioned at the LBB area. However, if a retro His potential is recorded from the prepositioned HB catheter during LBB area pacing it is possible to prove the engagement of the LBB by the pacing lead. The distance from the pacing spike to retro His plus pacing spike to the beginning of earliest surface QRS if was equivalent to baseline His to the beginning of earliest surface QRS interval, indicated evidence of LBB engagement

**FIGURE 7:**
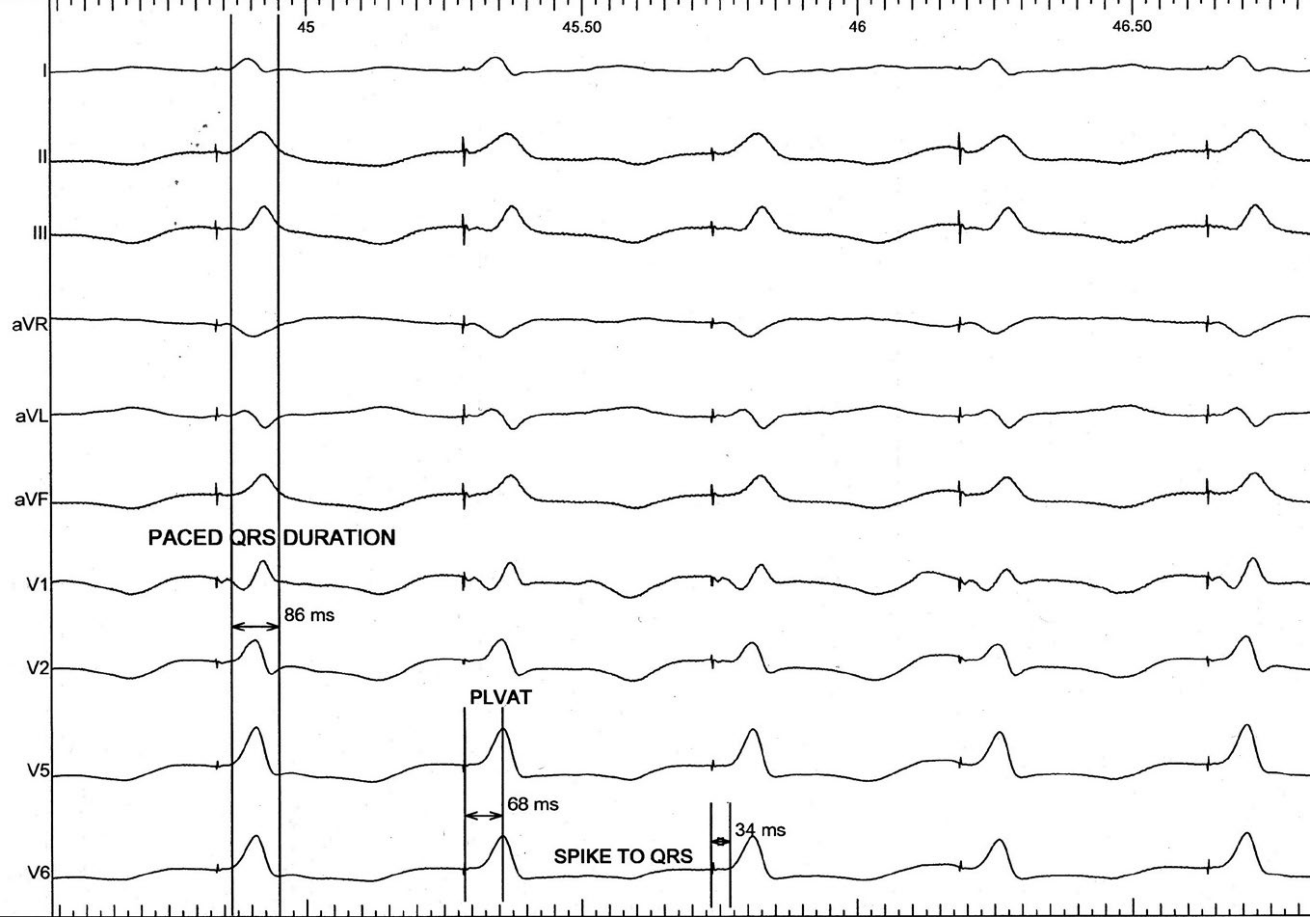
Measurement of different intervals in a patient with LBB area pacing

In cases of LBB area pacing stimulus to earliest surface QRS should be ideally equivalent (<10 ms variability) to baseline P‐ potential (LBB potential recorded from the pacing lead) to the earliest surface QRS when the recording was available. Normally the P‐ potential to earliest surface QRS interval is a short (≤30 ms) isoelectric segment. Usually, in patients with LBBB, this potential is not recordable. However, alternatively LBB captures during LBB area pacing can be ascertained in cases where it is not recordable. The sum of stimulus to retrograde His interval and stimulus to earliest surface QRS interval should be lesser than or equal to baseline His to earliest surface QRS interval (Figure 6). Peak LV activation time (pLVAT) is a measurement of local ventricular myocardial depolarization time and is measured as the interval from pacing stimulus to the peak of R wave in lead V4‐6. In patients with LBB area pacing remains very short (60‐90 ms) (Figure 7).

### 4.6 Selective vs non‐selective

There are no unified criteria to differentiate between selective and non‐selective LBB area pacing. If paced QRS duration and pLVAT in different pacing outputs remained unaltered, the pacing was considered selective. If pLVAT was prolonged during low output (1.0 V) compared to high output (5.0 V), it suggested the lead was closer to the LBB but not directly on it, hence a nonselective one. However, to avoid this we measured it at the high output in all patients.

### 4.7 Limitations

The major limitation of the present study was the relatively small sample size. So, the results may not be generalized. Secondly, hemodynamic response and clinical outcomes evidence were not measured in our study. The definition of successful LBB area pacing in our study was not on the EP features (ie, recording of LBB potential, getting different VA intervals during unipolar pacing from cathode and anode with 1:1 VA conduction, or finding different refractory periods with pacing by extra‐stimulus technique from cathode and anode). Retrograde His potential would be recorded better in narrowly spaced multipolar catheter with a smaller electrode. We have checked the ECG features in high output which may be altered in lower pacing output. However, standard setting of pacing output (safety margin of 3 times of threshold) was usually close to high output.

## 5 CONCLUSION

LBB area pacing results in narrower‐paced QRS complex in comparison to conventional RV apical pacing. The most common pattern of paced QRS complex in lead V1 was qR or Qr pattern and less commonly rSr’ or QS pattern. Patients with baseline RBBB showed lesser paced QRS shortening in comparison to patients with baseline LBBB.

## CONFLICT OF INTEREST

Authors declare no conflict of interests for this article.

## ETHICAL APPROVAL

IRB approval no. IPGME&R/IEC/2018/050, approval date: 20.01.2018.
